# Analysing comfort with primary care discussions and openness to social prescribing as mediators of the associations between loneliness and wellbeing among Canadians aged 55 and older

**DOI:** 10.1186/s12875-025-03067-7

**Published:** 2025-11-28

**Authors:** Daniel R. Y. Gan, Vivian Welch, Paul Hébert, Michelle Nelson, Kate Mulligan, Adam S. Hoverman, Sandra Allison, Grace Park, Kiffer G. Card

**Affiliations:** 1 Faculty of Health Sciences, Simon Fraser University, Burnaby, Canada; 2 Ministry of Health, Government of British Columbia, Victoria, Canada; 3 Bruyère Health Research Institute, University of Ottawa, Ottawa, Canada; 4 Centre Hospitalier de l’Université de Montréal, Montreal, Canada; 5 Dalla Lana School of Public Health, University of Toronto, Toronto, Canada; 6 Canadian Institute for Social Prescribing, Canadian Red Cross, Toronto, Canada; 7 Faculty of Medicine, University of British Columbia, Vancouver, Canada; 8 School of Public Health, University of Washington, Seattle, USA; 9 Canadian Alliance for Social Connection and Health, Victoria, Canada; 10 Pacific Regional Centre for Healthy Aging, Surrey, Canada; 11 Frasaer Health Authority, Surrey, Canada

**Keywords:** Integrated care, Gerontology, Health communication, Social isolation, Public health

## Abstract

**Background:**

Addressing the complex health and wellbeing challenges of older adults is a critical public health priority as populations age. Social Prescribing (SP) represents a promising strategy, connecting patients to non-clinical, community-based resources to enhance physical, mental, and social wellbeing.

**Methods:**

To develop a SP theory of change, this study used cross-sectional data from 2,450 community-dwelling older adults who participated in a population survey. Factor analyses identified four factors of comfort with primary care discussions (general, mental, physical, and social wellness) and three factors of openness to SP (effectiveness, meaningfulness, and supportiveness). Path analysis was conducted for each set of mediators separately.

**Results:**

Path analyses revealed that comfort with primary care discussions about *social wellness* (β = 0.08**) is associated with better wellbeing. People who report social loneliness are most comfortable with primary care discussions about *general wellness* (β = − 0.17***) and least comfortable with primary care discussions about *mental wellness* (β = − 0.24***), whereas people who report emotional loneliness are more likely to have similar levels of comfort to discuss *general wellness* and *mental wellness* (β = − 0.18***; − 0.18***). In addition, social loneliness is associated with less comfort with primary care discussions about *social wellness* (β = − 0.19***) and *mental wellness* (β = − 0.19***), whereas association is not found for emotional loneliness. These suggest that addressing the SP needs of people who experience emotional loneliness requires a different strategy. Reporting emotional loneliness is associated with *expressing support for SP* (β = 0.14***), which may be key to improving wellbeing (β = 0.10***) among this population. Overall, social loneliness has a total effect size of β_total_ = − 0.19, whereas emotional loneliness has a total effect size of β_total_ = − 0.45, more than 2.3 times larger.

**Conclusions:**

While SP may be acceptable to those who need it, some may experience greater difficulties accessing SP through primary care providers without interventions tailored to their loneliness status that could elicit buy-in and enrolment. Primary care providers may wish to pay closer attention to people with emotional loneliness. Other considerations, such as trust and motivational interviewing for positive self-beliefs may explain potential changes from loneliness to wellbeing.

## Background

As populations age globally, the wellbeing of older adults has emerged as a critical public health priority. Older adults often face multifaceted challenges that impact their physical, mental, and social health, including chronic illnesses, social isolation, and limited access to supportive services. Addressing these complex needs requires innovative approaches that extend beyond traditional medical care. One such approach gaining attention is Social Prescribing (SP), a holistic strategy that connects patients from various clinical touchpoints such as primary and community care [1] with non-clinical community resources to enhance their overall wellbeing.

Social Prescribing can play a pivotal role in supporting older patients by facilitating access to various community-based activities and services. These may include engagement in social clubs, physical exercise programs, mental health support groups, and volunteer opportunities. By leveraging these resources, SP aims to address social isolation as a determinant of health, thereby improving older adults’ quality of life, reducing loneliness, and mitigating the effects of chronic conditions [2, 3]. The integrative nature of SP aligns with the growing recognition that health is not solely the absence of disease but “the ability to adapt to one’s environment” for “full, fruitful creative living” which encompasses overall wellbeing [4, 5], including emotional and social dimensions. Because medical consultations traditionally focus on physical health, some older adults may feel uncomfortable addressing mental health issues or social challenges with their primary care providers [6]. Recognizing and addressing these discomforts is crucial to increase SP enrolment and improve follow through with better communication during primary care.

Besides comfort with primary care discussions, understanding older adults’ attitudes toward SP is important for the successful implementation and scalability of SP programs. Older adults’ perceptions of activity effectiveness, the meaningfulness of the interventions, and their willingness to support such initiatives can significantly influence participation rates and outcomes [7, 8]. These perceptions and any discomfort with primary care may be influenced by chronic loneliness that may have developed over time [9]. Older adults who experience loneliness and chronic illnesses, mental health challenges, or lower levels of social connection may have unique reservations that reduce their openness to SP and comfort with primary care discussions. Recognizing the dimensions and compounding influence of loneliness may help to develop targeted interventions that address common concerns and optimize the reach and effectiveness of SP programs. This study aims to develop a SP theory of change from loneliness to wellbeing by exploring possible levers of social and emotional processes in primary care settings [10].

Broadly, current literature distinguishes loneliness from social isolation. Whereas social isolation depends largely on the number of persons one comes into (various forms of) contact with, loneliness is primarily a feeling of inadequate social connectedness which has emotional and social dimensions [11]. High levels of loneliness may tap onto an unshared, ungenerative, and decaying state of mind [12]. Social and emotional dimensions of loneliness correspond to unmet relational and intimacy needs [11, 13]. While social loneliness may be understood quite simply as (uncountable but measurable) perceptions of inadequate social connections (countable, however defined) and its influences on wellbeing outcomes have been successfully explained through mediation analyses [14], emotional loneliness is more complex and less well understood. Variables and circumstances such as rejection among formerly married older adults are particularly salient for social loneliness [9], but the same cannot be said of emotional loneliness.

Emotional loneliness is associated with diverse experiences of close relationships and negative life events [15, 16]. Living without a partner is associated with increased emotional loneliness [16]. Among people who are partnered, women were more likely to report emotional loneliness than men [16]. Being partnered decreases social loneliness in men but not women [16]. Similarly, psychological distress fully explained associations between social loneliness and sleep difficulties but only partially explained associations between emotional loneliness and sleep difficulties [14]. People who experience emotional loneliness may be more prone to rumination [17], but mediation studies of its mechanisms of action are in nascent stages. Together, these studies led to our analytical framework to explore how emotional loneliness is qualitatively different and more complex, and may require a different strategy to address than social loneliness.

Personal depth and emotional tact to offer care and broach complex topics of maladaptive beliefs may be necessary to address emotional loneliness due to spousal relationships or loss of confidants [18, 19]. To illustrate, emotional loneliness may stem from unmet expectations of emotional support from intimate relationships in later life, such that one is “keenly distressed when it fails to materialize. Similarly, sharing a household with a spouse who is not emotionally supportive. may be particularly distressing because seeking to avoid or distance oneself from a spouse” seems challenging [9]. Emotional loneliness arising from such negative incidents in intimate relationships may cause one to adopt ruminative patterns and negative self-beliefs over time [16], possibly reinforcing notions of helplessness and hopelessness [20]. While existential loneliness (i.e., a lack of life-meaning) may be conceptually distinct [21], its emphasis on a sense of emptiness [22] overlaps with emotional loneliness and stands in contrast to successful SP programs that emphasize motivational interviewing, communal activities, and socio-ecological understandings of persons [19].

Building on the foundational understanding of older adults’ wellbeing and their interactions with primary care [7], this study explores how primary care providers may broach the topic of Social Prescribing in supporting older adult populations who experience loneliness. Specifically, it seeks to identify relevant factors of comfort with primary care and openness to SP, and examine their mediating influences on the relationships between loneliness and wellbeing in response to calls from a systematic review to examine pathways [23]. Furthermore, this study aims to develop theoretical models of change that reflect barriers older adults face with varying levels of loneliness. Through factor and path analyses, the study will inform future interventions and guidelines for enrolling older adults who experience loneliness into SP at primary care settings. Ultimately, better communication between older adults and primary care providers will foster healthier, more connected, and fulfilled older adult communities.

## Methods

### Study design

A cross-sectional survey of Canadian adults aged 55 and older was conducted and analyzed using psychometrically validated measures. We conducted purposive sampling with the aim of approximating general community-dwelling older adult populations, i.e., with a mix of health status and service utilization patterns [24]. Details of the overall survey participants and measures used have been published elsewhere [24].

### Participants and recruitment

Participants were Canadian adults aged 55 years and older who were able to respond to a digital survey, recruited through various online and in-person channels to ensure broad representation and demographic diversity during the summer of 2022. Recruitment strategies included paid online advertisements, where targeted ads were placed on major social media platforms such as Facebook, Instagram, and X (Twitter), as well as through Google Ads, designed to reach a wide audience of older adults and encourage participation in the survey. Additionally, we engaged front-line healthcare services, including Red Cross, United Way, clinics and non-profit organizations engaged in SP, to distribute survey invitations, leveraging their direct contact with the older adult population to enhance participation rates.

Furthermore, community email outreach comprised additional paid advertisements and email campaigns to reach underrepresented demographic groups, thereby increasing the diversity of the participant pool. Recruitment continued until we obtained a predetermined minimum number of older adults in subgroups of interest (e.g., income). In the process of increasing representation from an underrepresented group (e.g., through targeted invitations), we did not exclude interested participants by other categories (e.g., gender), resulting in the overrepresentation of some categories (e.g., women).

### Consent and survey procedure

Interested individuals recruited as described above accessed the online survey via a link provided through the recruitment channels. The survey was hosted on Qualtrics, a secure digital survey platform. Upon accessing the survey, participants were given an informed consent form detailing the study’s purpose, procedures, confidentiality measures, and their rights as participants. Only those who provided informed consent were permitted to proceed with the survey. Respondents were automatically entered into a prize draw with a total prize of $200 CAD cash to encourage participation. Each participant had a 1 in 100 chance of winning the prize, contingent upon survey completion.

### Measures

#### Comfort with Primary Care Discussions

Participants were asked “How comfortable are you with your healthcare provider or someone working at your local health clinic doing each of the following?” in relation to a list of >14 items [24]. Examples include “Giving you advice about social connection or relationships,” “Giving you a referral to meet nutritionist,” and “Giving you a referral to participate in a community organization.” Responses were rated on a Likert scale from 1 (not at all comfortable) to 5 (very comfortable), with higher scores indicating greater comfort.

#### Openness to social prescribing

The survey described SP and participants were asked to “help us understand your attitudes” toward SP by indicating their level of agreement with 11 items [24]. Examples include “SP is a good way to address my needs,” “Money from the government used to facilitate SP programs would be money well spent,” and “I would participate in a SP program.” Responses were rated on a Likert scale from 1 (strongly disagree) to 5 (strongly agree), with higher scores indicating greater openness to SP.

#### Wellbeing

Wellbeing was measured using 2-items instruments of self-rated health, anxiety, and depressive symptoms. For self-rated health, participants were asked to rate their physical and mental health using two single-item measures [25, 26], with responses ranging from 1 (poor) to 5 (excellent). For anxiety symptoms, participants were asked how often they “felt nervous, anxious or on edge” or were “not able to stop worrying or control your worries” in the past two weeks using the 2-item Generalized Anxiety Disorder Scale (GAD-2) [27], with responses ranging from 0 (not at all) to 3 (nearly every day). For depressive symptoms, participants were asked how often they experienced “little interest or pleasure in doing things” or “feeling down, depressed or hopeless” in the past two weeks using the 2-item Patient Health Questionnaire (PHQ-2) [28], with responses ranging from 0 (not at all) to 3 (nearly every day). Items for anxiety and depressive symptoms were reverse-coded such that higher scores indicated less symptoms. A standardized mean score was generated and the scale items had good internal consistency (Cronbach’s α = 0.839).

#### Loneliness

Loneliness was measured using the six-item De Jong-Gierveld Loneliness Scale (DJGLS-6) with a standard score of 3 or more indicating being lonely [11]. Loneliness comprised two factors in the path analyses. For emotional loneliness, participants were asked whether they “experience a general sense of emptiness,” “often feel rejected,” and “miss having people around,” with responses ranging from 0 (no) to 0.5 (more or less) to 1 (yes). For social loneliness, participants were asked whether there are “plenty of people I can rely on when I have problems,” “many people I can trust completely,” and “enough people I feel close to,” with responses ranging from 0 (no) to 0.5 (more or less) to 1 (yes). Items for social loneliness were reverse-coded such that higher scores indicated more loneliness. Mean scores were generated for both factors (Cronbach’s α = 0.699, 0.841).

#### Sociodemographics

Age was measured as an ordinal variable with 5-year age intervals from 1 (high 50 s) to 9 (high 90 s). Gender was measured by asking participants whether they identified as (1) a woman (including transwoman) or (0) other options, namely a man (including transman) or non-binary person (including agender, genderfluid, or genderqueer), and was dichotomized for the purposes of this analysis. Household income was measured as an ordinal variable with intervals of $5,000 up to $50,000 and intervals of $10,000 thereon until “$200,000 or more.” Responses were recategorized into 4 groups: “Under $30,000”, “$30,000 to $59,999”, “$60,000 to $99,999”, “$100,000 or more” to approximate normal distribution.

### Statistical analysis

All analyses were conducted using Stata. Missing data were handled using listwise deletion. Analyses proceeded in two stages, focusing on a factor analysis followed by a path analysis.

#### Factor Analysis

Exploratory Factor Analysis (EFA) with varimax rotation was conducted to explore the underlying structure of *comfort with primary care discussions* and *openness to SP*. Items were grouped based on factor loadings, and internal consistency for each factor was assessed using Cronbach’s alpha. This step allows the creation of psychometrically validated factors that capture distinct aspects of *comfort with primary care discussions* and *openness to SP* for further analysis. Unstandardized item means were calculated for the identified factors to facilitate interpretation and comparison of results across analyses.

#### Path Analysis

Path analysis was conducted to develop a theory of change from loneliness to wellbeing through factors of *comfort with primary care discussions* and *openness to SP*. Path analysis through these factors were conducted in two separate models. All main variables were allowed to be correlated. Mediating and outcome variables were controlled for sociodemographic variables if they were significantly correlated at zeroth order. Statistically significant paths and beta coefficients were indicated with *p <.05, **p <.01, or ***p <.001. Goodness-of-fit indices, including the Comparative Fit Index (CFI), Tucker-Lewis Index (TLI), and Root Mean Square Error of Approximation (RMSEA), were used to evaluate model fit [29]. Sensitivity analysis was conducted by removing any collinear mediators.

Indirect effect sizes were calculated as a product of beta coefficients along statistically significant paths from loneliness to wellbeing. For each full model, indirect effect sizes were summed to compute the percentage of mediated effects as a fraction of total effects.

### Ethical considerations

The study was conducted in accordance with ethical guidelines for research involving human participants. All participants provided informed consent prior to participation, and data were collected anonymously to protect participant confidentiality. The study protocol was reviewed by the Research Ethics Board at Simon Fraser University.

## Results

This study used data from 2,450 participants, part of a larger full sample comprising more than 4,100 participants who responded to the survey on Social Prescribing Needs of Older Adults in Canada [24]. The average age of our sample was 69.5 years old, with 74.7% women and 65.8% reported less than $59,999 annual income (see Table 1 for details). Approximately 34.7% reported having chronic illness(es), and 42.8% were lonely, i.e., scored 3 or more on DJGLS-6. Participants were randomly allocated to answer questions on comfort with primary care discussions (subsample 1) or openness to SP (subsample 2). We present results from factor analyses followed by path analyses. Given the exploratory nature of this study to develop theoretical models of change for future intervention development, we identify relevant propositions throughout the results section for subsequent discussions.

**Table 1:** Descriptive statistics of subsamples used in analysis after listwise deletion (*n* = 2450)

	Subsample 1 (*n* = 1282)				Subsample 2 (*n* = 1168)			
	Mean	SE	95% Confidence Interval		Mean	SE	95% Confidence Interval	
Control variables:								
Age (years)	69.8	0.220	69.4	70.2	69.2	0.237	68.8	69.7
Women	73.7%	0.012	0.713	0.761	75.7%	0.013	0.732	0.781
Income group	2.196	0.028	2.140	2.251	2.180	0.030	2.121	2.239
Chronic illness (binary)	35.3%	0.013	32.7%	38.0%	34.0%	0.014	31.2%	36.7%
Lonely (DJGLS ≥ 3; binary)	41.8%	0.014	39.1%	44.6%	43.8%	0.015	40.9%	46.8%
Social loneliness	0.474	0.010	0.455	0.493	0.490	0.010	0.469	0.510
Emotional loneliness	0.331	0.008	0.314	0.347	0.331	0.009	0.313	0.349
Comfort with primary care discussions (CPD) on:								
Social wellness	3.579	0.034	3.511	3.646	-	-	-	-
Mental wellness	4.031	0.027	3.977	4.085	-	-	-	-
Physical wellness	3.991	0.028	3.936	4.047	-	-	-	-
General wellness	4.307	0.025	4.258	4.355	-	-	-	-
Openness to SP as demonstrated by:								
Beliefs in SP effectiveness	-	-	-	-	3.316	0.025	3.267	3.365
Expressing support for SP	-	-	-	-	3.515	0.027	3.462	3.568
Beliefs in SP meaningfulness	-	-	-	-	3.702	0.023	3.657	3.746
Wellbeing	0.472	0.021	0.430	0.514	0.489	0.022	0.446	0.532

### Factors of comfort with primary care discussions and openness to SP

Comfort with primary care discussions comprises four factors, namely comfort with primary care discussions about general, mental, physical, and social wellness (see Table 2 for factor loadings). We use “wellness” to emphasize health service users’ perspectives [30]. Cronbach’s alpha indicated good reliability for all factors with item factor loadings exceeding 0.51. *Comfort with primary care discussions aboutgeneral wellness* includes 4 items, such as asking about “physical activity,” “diet,” and substance use (Cronbach’s α = 0.868). *Comfort with primary care discussions aboutmental wellness* includes 5 items such as giving advice about “mental health,” “social connection or relationships,” and “what activities to do for fun” (Cronbach’s α = 0.901). *Comfort with primary care discussions aboutphysical wellness* includes 3 items on giving referrals to meet a “physical trainer,” “nutritionist,” and “physical therapist or physiotherapist” (Cronbach’s α = 0.836). *Comfort with primary care discussions aboutsocial wellness* includes 2 items on giving referrals to “participate in” and “volunteer for” a community organization (Cronbach’s α = 0.890).

**Table 2:** Rotated factor loadings of items comprising comfort with primary care discussions

Item	Factor loadings				Uniqueness
	Mental wellness	General wellness	Physical wellness	Social wellness	
Asking you about your mental health	**0.80**	0.55	0.17	0.16	0.31
Giving you advice about social connection or relationships	**0.70**	0.25	0.23	0.38	0.22
Giving you advice about mental health	**0.67**	0.37	0.23	0.24	0.22
Asking about social connection or your relationships	**0.62**	0.46	0.16	0.24	0.29
Giving you advice about what activities to do for fun	**0.61**	0.22	0.18	0.43	0.32
Asking you about physical activity	0.27	**0.78**	0.22	0.17	0.23
Asking you about your diet	0.25	**0.76**	0.19	0.16	0.27
Giving you advice about physical activity	0.48	**0.55**	0.27	0.25	0.25
Asking you about using alcohol, tobacco, or other drugs	0.18	**0.51**	0.11	0.15	0.42
Giving you a referral to meet a physical trainer	0.23	0.28	**0.60**	0.38	0.37
Giving you a referral to meet nutritionist	0.32	0.28	**0.58**	0.31	0.35
Giving you a referral to meet a physical therapist or physiotherapist	0.22	0.32	**0.57**	0.30	0.41
Giving you a referral to participate in a community organization	0.25	0.17	0.24	**0.79**	0.23
Giving you a referral to volunteer for a community-organization	0.24	0.15	0.17	**0.78**	0.28
**Alpha (bolded items only)**	**0.901**	**0.868**	**0.836**	**0.890**	

Openness to SP comprises three factors, namely *beliefs about SP effectiveness*, *beliefs about SP meaningfulness*, and *expressing support for SP* (see Table 3 for factor loadings). Cronbach’s alpha indicated good reliability for all factors with item factor loadings exceeding 0.55. *Beliefs about SP effectiveness* include 5 items such as “SP is a good way to address my needs” and “I would benefit from participating in a SP program” (α = 0.931) with higher scores indicating more positive beliefs about SP. *Beliefs about SP meaningfulness* include 4 items such as “SP would help connect people and communities” and “Money from the government used to facilitate SP programs would be money well spent” (α = 0.865) with higher scores indicating more positive beliefs about SP. *Expressing support for SP* includes 2 items, namely “I would participate in a SP program” and “I would approve of my healthcare provider using SP” (α = 0.833) with higher scores indicating more expressed support for SP.

**Table 3:** Rotated factor loadings of items comprising openness to SP

Item	Factor loadings			Uniqueness
	Effectiveness	Meaningfulness	Supportiveness	
SP is a good way to address my needs.	**0.80**	0.33	0.25	0.18
I would benefit from participating in a SP program.	**0.78**	0.34	0.23	0.23
SP seems like it would meet my needs.	**0.69**	0.28	0.43	0.25
I could participate in SP if my healthcare provider offered it.	**0.61**	0.38	0.44	0.27
I would follow through on a recommendation from my doctor to participate in SP.	**0.60**	0.35	0.41	0.31
SP would help connect people and communities.	0.33	**0.72**	0.26	0.31
Money from the government used to facilitate SP programs would be money well spent.	0.41	**0.70**	0.23	0.30
SP sounds like it would help a lot of people.	0.32	**0.57**	0.33	0.46
SP would help show that my healthcare provider cares about me.	0.45	**0.55**	0.22	0.44
I would participate in a SP program.	0.54	0.30	**0.61**	0.24
I would approve of my healthcare provider using SP.	0.40	0.34	**0.57**	0.39
**Alpha (bolded items only)**	**0.931**	**0.865**	**0.833**	

Unstandardized item means were calculated for identified factors and were used in subsequent analyses to examine their mediating influences from loneliness to wellbeing.

### Mediating influences

Two structural equation models (SEM) were examined to understand how factors of comfort with primary care discussions and openness to SP might mediate the relationship between loneliness and wellbeing. Wellbeing, *comfort with primary care discussions about mental* and *general wellness* were controlled for age, gender, and income. *Comfort with primary care discussions about social wellness* and *physical wellness* were controlled for age and income. *Beliefs about SP effectiveness* were controlled for gender and income. *Beliefs about SP meaningfulness* and *expressing support for SP* were controlled for gender. Both models showed excellent fit based on goodness-of-fit indices (Model 1: CFI = 1.000, TLI = 1.003, RMSEA = 0.000, SRMR = 0.006; chi-square p-value = 0.475; AIC = 25220, BIC = 25441; Model 2: CFI = 0.999, TLI = 0.997, RMSEA = 0.017, SRMR = 0.006; chi-square p-value = 0.239; AIC = 18593, BIC = 18740).

**Fig. 1:**
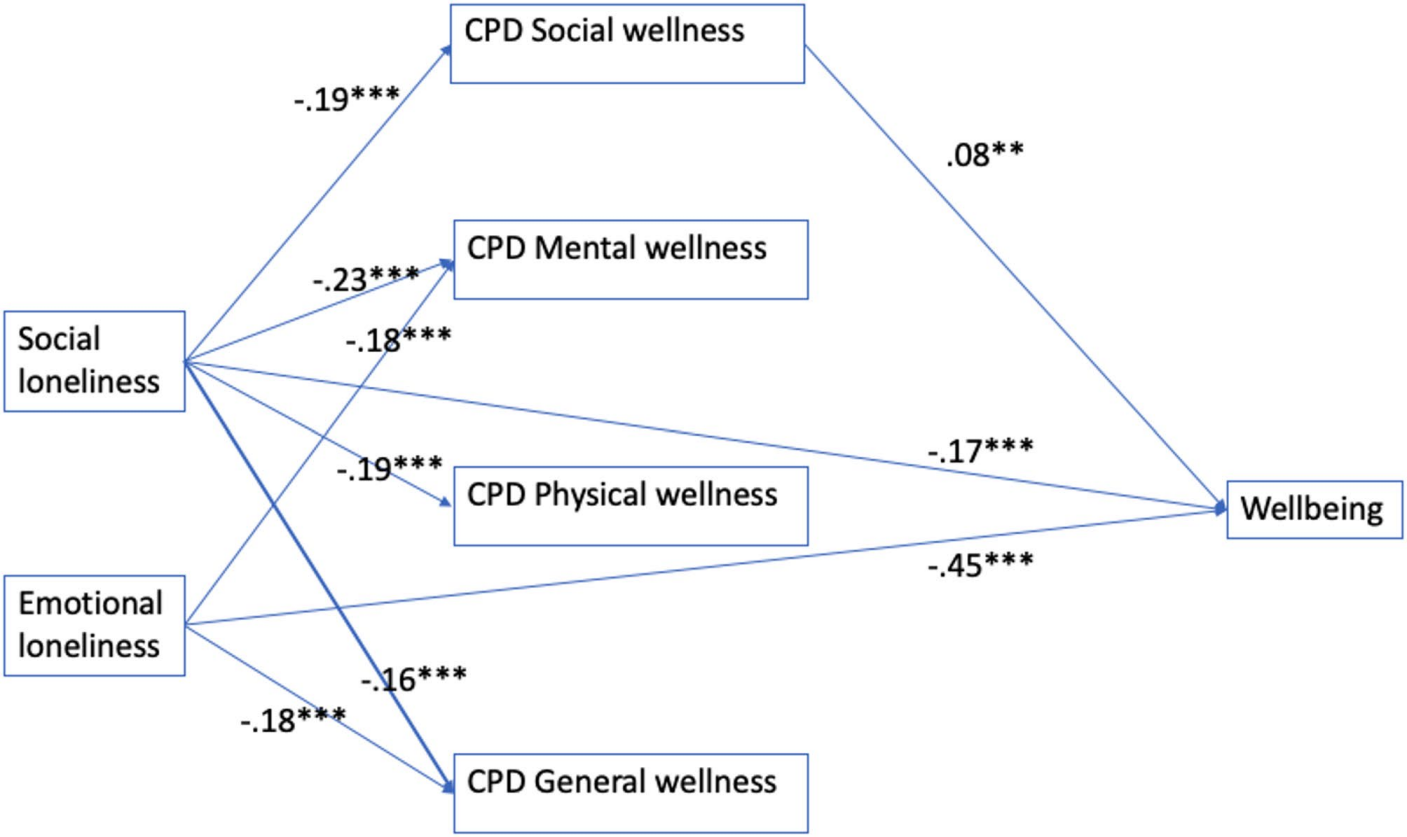
Model 1 showing paths from loneliness to wellbeing (outcome) through comfort with primary care discussions (CPD) factors (from factor analysis)

**Fig. 2:**
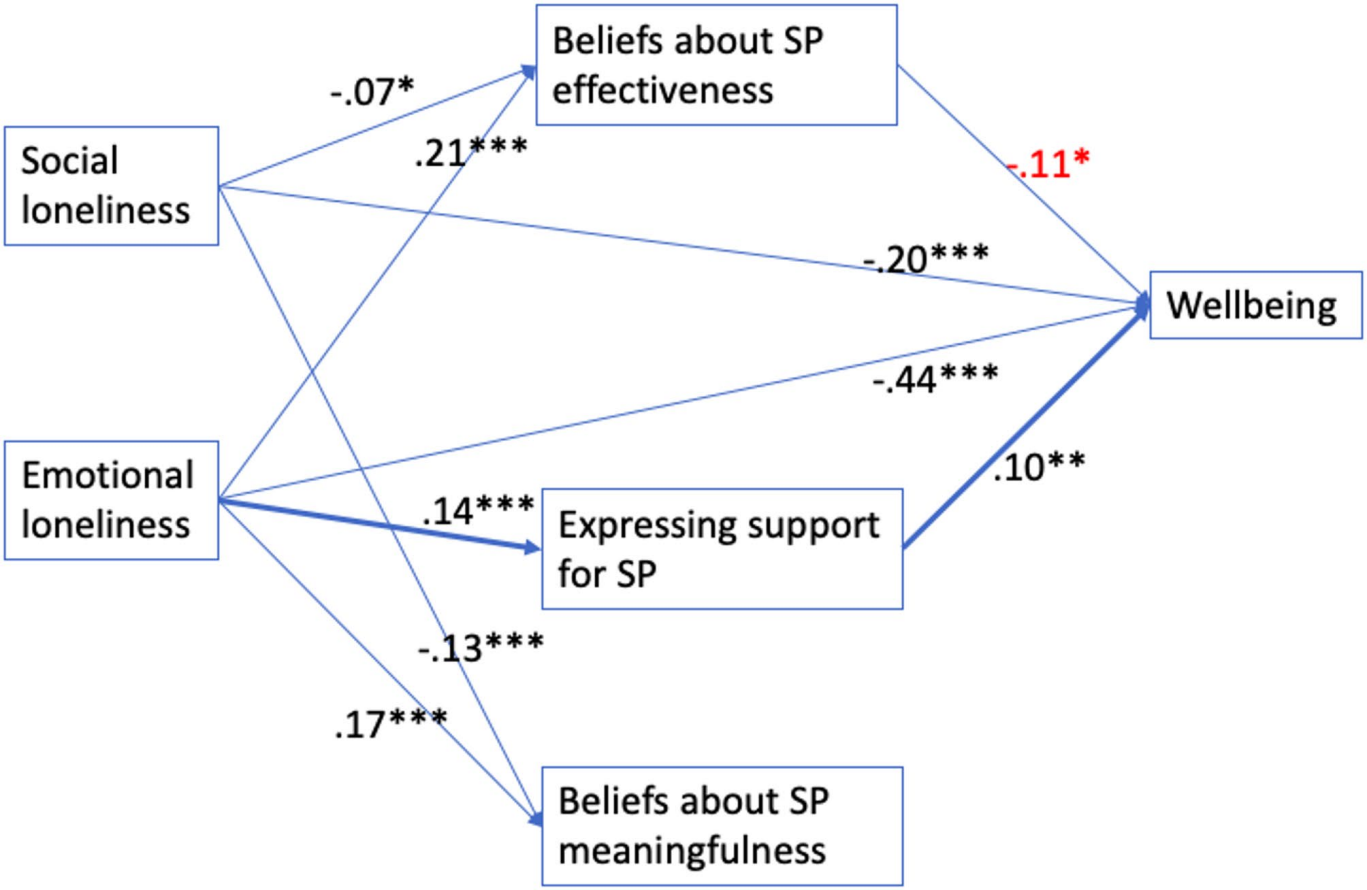
Model 2 showing paths from loneliness to wellbeing (outcome) through openness to social prescribing (SP) factors (from factor analysis)

Based on the model in Fig. 1 (*n* = 1,282), *comfort with primary care discussions about social wellness* is associated with better wellbeing (β = − 0.08**). People who report social loneliness are most *comfortable with primary care discussions about general wellness* (β = − 0.16***) and least *comfortable with primary care discussions about mental wellness* (β = − 0.23***), whereas people who report emotional loneliness are more likely to have similar levels of *comfort to discuss general wellness* and *mental wellness* (β = − 0.18***; − 0.18***). In addition, social loneliness is also associated with less *comfort with primary care discussions about social wellness* (β = − 0.19***) and *physical wellness* (β = − 0.19***), whereas associations are not found for emotional loneliness. Overall, these suggest that primary care providers may focus on discussions about *social wellness* given its potential to lead to wellbeing (Proposition 1), and may benefit from further strategizing around the SP needs of people who experience emotional loneliness given unknown comfort or discomfort discussing social wellness (i.e., no paths from emotional loneliness to *comfort with primary care discussions about social wellness*) which brings us to Fig. 2.

Based on the model in Fig. 2 (*n* = 1168), people who experience emotional loneliness are more likely to hold positive *beliefs about SP effectiveness* (β = 0.21***) and *meaningfulness* (β = 0.17***), whereas people who experience social loneliness are less likely to hold positive *beliefs about SP meaningfulness* (β = − 0.13***) and *effectiveness* (β = − 0.07*). In addition, reporting emotional loneliness is associated with *expressing support for SP* (β = 0.14***), which may be key to improved wellbeing (β = 0.10***) in this population (Proposition 2).

*Beliefs about SP meaningfulness* are not associated with wellbeing. Surprisingly, positive *beliefs about SP effectiveness* are associated with poorer wellbeing (β = − 0.11*) in the full model (Fig. 2). After removing collinear variable *beliefs about SP effectiveness* in the sensitivity analysis (model not shown), *expressing support for SP* is no longer associated with wellbeing, but other associations remain.

In both mediation models, wellbeing remains directly associated with social loneliness (β = − 0.17***; − 0.20***) and emotional loneliness (β = − 0.45***; − 0.44***), suggesting that there are other mediators not included in this study (e.g., trust) that may explain the relationship between loneliness and wellbeing (Proposition 3).

### Effect size analysis

In Model 1, indirect effects from social loneliness to wellbeing is β_indirect_ = -(.19x.08) = − 0.0152 which constitutes − 0.0152/(−0.0152-0.17) = 8.2% of the total effects of social loneliness on wellbeing. In comparison, there are no indirect effects from emotional loneliness to wellbeing such that β_indirect_ = 0 constitutes 0/(−0.45) = 0% of the total effects of emotional loneliness on wellbeing. This means that Model 1 better explains the influences of social loneliness compared to the influences of emotional loneliness.

In Model 2, indirect effects from social loneliness to wellbeing is β_indirect_ =.07x.11 = 0.0077 which constitutes 0.0077/(0.0077–0.20.0077.20) = − 4.0% of the total effects of social loneliness on wellbeing. Indirect effects from emotional loneliness to wellbeing is β_indirect_ = -(.21x.11)+(.14x.10) = − 0.0091 which constitutes − 0.0091/(−0.0091-0.44) = 2.0% of the total effects of emotional loneliness on wellbeing. This shows that Model 2 better explains the influences of emotional loneliness compared to Model 1.

Overall, social loneliness has total effect sizes (β_total_) ranging from − 0.0152 to 0.17 = − 0.1852 (Model 1) to 0.0077–0.20.0077.20 = − 0.1923 (Model 2), whereas emotional loneliness has total effects sizes (β_total_) ranging from − 0.45 (Model 1) to − 0.0091–0.44.0091.44 = − 0.4491 (Model 2), which are approximately (−0.45-0.4491)/(−0.1852-0.1923) = 2.38 times larger than those of social loneliness. This means that primary care providers may wish to pay closer attention to emotional loneliness (Proposition 4).

## Discussion

This study explored comfort with primary care discussions and openness to Social Prescribing (SP) as a means of enhancing wellbeing among older adults who experience loneliness. Findings suggest that older adults’ *comfort with primary care discussions about social wellness* and *expressing support for SP* are associated with better wellbeing. Path analytic models showed that social and emotional loneliness influence comfort with primary care discussions and openness to SP differently, and may thus require different approaches to support SP enrolment. Overall, emotional loneliness is especially detrimental to wellbeing. Model 1 presents ways forward to better wellbeing among people who experience social loneliness, whereas Model 2 suggests ways forward among people who experience emotional loneliness. The following paragraphs discuss four propositions in relation to existing literature before turning to implications for practice and research. The propositions for discussions have been rearranged for flow.

### Primary care discussions may focus on social wellness

Our analyses in Model 1 suggest that *comfort with primary care discussions about social wellness* may be key to improving wellbeing among people with social loneliness. This is congruent with effective interventions (such as horticulture and walking groups) that highlighted social interactions as an important mediator of the effects of active living [31–33]. As a first entry point to health systems, primary care interactions that focus on wellbeing, including social wellbeing, can be deeply transformational to health services delivery [34, 35]. Transformative service researchers have asked: “What impact do generalized best practices have on the well-being of individual consumers? … What aspects of healthcare offerings, service models, or service designs help reduce health disparities?” [34]. To date, service delivery innovations include using a stepped logic to involve social professionals [35] and using academy-community partnerships to integrate wellness program delivery in high-needs settings with student training [19].

Examples of focusing on social wellness include forming and sustaining interprofessional teams to deliver regular wellness clinics at supportive housing or primary care centres [19, 36]. After identifying root causes of new issues, community members were reminded of communal programs and “social activities to reduce social isolation, and create a sense of community” [19]. Holistic, socio-ecological understanding of patients’ lives, including “social isolation from lack of transportation, neighborhood violence, and other family-based factors” led to “trust and rapport” and required training in “motivational interviewing, holistic assessment, TEAMSTEPPS, and … age, cultural, and literacy-level appropriate” education [19].

### Emotional loneliness is 2.38 times more detrimental on wellbeing than social loneliness

Our effect size analyses suggest that focused attention to emotional loneliness may be warranted. Of the two forms of loneliness, emotional loneliness is more detrimental and more difficult to resolve [37]. This is congruent with older adult studies (age 70+) that reported 1.39/0.95 = 1.46 to 3.73/1.84 = 2.02 times the impact of emotional loneliness on physical and mental health-related quality of life (HRQoL) than social loneliness [38]. Some studies even found low levels of social loneliness among older adults (age 60+) to be protective [15, 39], perhaps because it indicates self-awareness and acceptance toward action. A Dutch study reported stable levels of social loneliness but increasing emotional loneliness during the COVID-19 pandemic [37]. These point to the importance of addressing emotional loneliness, especially among older adults. Among young adults (age 21–35), social loneliness may be more impactful, with (2.69-1.69)/(2.33-1.33) = 1.24 times the impact on sleep difficulties as compared to emotional loneliness, or (1.46-1.46)/(1.22-1.22) = 2.09 times after adjusting for anxiety and depression [14]. In other words, the significance of emotional loneliness increases with age.

### *Expressing support for SP *may improve wellbeing among people who report emotional loneliness

Our analyses in Model 2 suggest that *expressing support for SP* may be a key to improved wellbeing among people with emotional loneliness. Psychologists have observed that “expression of negative feelings is adaptive to the extent that it leads to some kind of resolution involving the source or significance of distress” [40]. Among respondents who experience emotional loneliness, *expressing support for SP* may be understood as an act of acknowledging the possibility of change and self-advocacy toward positive resolution. This is consistent with studies that found self-efficacy as a mediator between loneliness and mental and physical health-related quality of life [41]. This acceptance of one’s needs and circumstances, and expressed willingness to take risk to form new friendships and move toward wellbeing is inherently transformative and salubrious based on our definition of health as adaptability or high change readiness [42].

*Expressing support for SP* is an important factor of openness to SP. Openness to changing deeply held (and previously confirmed) negative self-beliefs and finding (new) meaningful relationships has been described elsewhere as “unlocking a better sense of self for psycho-sociability” [43]. Clinical strategies of motivational interviewing with patients include “reflective listening and eliciting change talk … to work through their ambivalence” [44]. Knowing when and how to elicit patients’ support for SP is an important step that could help them move from pre-contemplative or contemplative stages toward greater change readiness [45]. Current primary care providers, like trainees of the community-based wellness clinic [22], will need training in motivational interviewing to elicit support for SP by communicating the appropriateness of SP “aims and projected outcomes” at the right juncture without arousing any resistance to change [45].

### Other variables may explain the relationship between loneliness and wellbeing

Overall, these findings align with prior research emphasizing the importance of provider-patient trust and holistic and patient-centred care approaches to successfully enrol patients in SP [46]. Positive associations between comfort in primary care discussions and better wellbeing (Model 1) corroborate studies that highlight the importance of addressing social and emotional needs at primary care touchpoints [47]. Furthermore, the positive association between SP supportiveness and wellbeing (Model 2) presents an avenue to improve wellbeing among people who experience emotional loneliness. While these insights supported data-informed propositions to develop SP theories of change, more than 90% of direct effects from loneliness to wellbeing remained in both models. This indicates that other variables may explain the relationship between loneliness and wellbeing. Based on the discussions and literature cited above, other mediators may include self-beliefs, self-efficacy, resilience, trust, change readiness, length of patient-provider relationship, and motivational interviewing skills of primary care providers. Including these variables alongside comfort and openness to SP in a single, more fulsome model may validate parts of the SP theories of change outlined above.

### Implications for practice

Taken together, the SP theories of change developed in this paper underscore the need for primary care providers to create an environment that encourages older adults to discuss health as adaptability for “full, fruitful creative living” [48], including social and emotional wellness. Developing tailored interventions and training in motivational interviewing that considers older adults’ social or emotional loneliness is crucial for optimizing SP enrolment. Training for healthcare professionals to enhance communication skills and reduce stigma around non-medical discussions could improve patient comfort. More research on why subpopulations experience discomfort talking about social and emotional wellness with their primary care providers may help. More systematic approaches to integrate SP into primary care practices may also help to address broader determinants of health with community resources [35].

In the interim, we highlight extant literature which recommends that clinicians ask patients, “What matters to you?” [49]. With reference to De Jong-Gierveld’s loneliness scale [11], we venture to recommend also asking: “Who do you rely on when you face problems?” and if patients reported relying on no one, to listen attentively and actively (reflecting to patients what we hear) and when appropriate follow-up with “What might you do if you experience a sense of emptiness?”. These open-ended questions follow motivational interviewing and coaching principles to activate the person’s agency to seek solutions. When there are some indicators that the patient is open to change and SP, it might then be appropriate to ask whether they would like to participate in a SP program and so elicit an agreement and buy-in that is already innate.

### Strengths and limitations

A key strength of this study is its systematic approach to evaluating dimensions of comfort with primary care and openness to SP, employing validated measures and robust statistical methods. The use of path analysis provided insights into direct and indirect relationships between variables, allowing useful propositions for theoretical developments to emerge. That said, the SP theories of change remain to be tested. The cross-sectional design limits causal inferences, and self-reported data may be subject to social desirability and recall biases. Additionally, while the study employed diverse recruitment strategies, the sample may not fully represent marginalized older adult populations, such as those in rural or low-income settings, with severe cognitive decline or other conditions that might preclude them from participating in a digital survey, which limits generalizability. The unique needs of subpopulations and variations across different socio-demographic groups, such as socio-cultural needs [50], will be explored in a separate study.

### Future research

Based on results, there would be a value in considering longitudinal dyadic designs to establish causality and examine the long-term impacts of SP interventions on health outcomes, including the measures of primary care providers. Research may also explore comfort with primary care discussions and openness to SP among underserved populations and develop culturally tailored approaches to address these barriers. Investigating the role of digital therapeutics and tools in facilitating changes in sense of self like reSET-O [51] and SP referrals, tracking, and monitoring may provide insights into scaling interventions for broader reach [31]. Additionally, studies capturing older adults’ preferences and lived experiences with SP can inform program development.

## Conclusion

This study highlights the significance of comfort with primary care discussions and openness to SP in promoting older adults’ health and wellbeing. By fostering a welcoming environment for wellness discussions and integrating motivational interviewing into care practices, primary care providers can optimize SP enrolment and address the multifaceted needs of older adults. Future efforts could focus on operationalizing SP theories of change with motivational interviewing training tailored to older populations with social and/or emotional loneliness, and leveraging integrated knowledge translation to enhance the reach and impact of the SP theories of change developed through systematic model building and testing. Together, these strategies hold promise for advancing SP implementation toward a goal of healthier and more connected communities and older adults.

## Data Availability

The datasets used during the current study are available from the corresponding author on reasonable request.

## Abbreviations

SP: Social prescribing
CPD: Comfort with primary care discussions
